# Exosomal long non-coding RNA LINC00662 promotes non-small cell lung cancer progression by miR-320d/E2F1 axis

**DOI:** 10.18632/aging.202522

**Published:** 2021-02-11

**Authors:** Xin Lv, Yingjie Lian, Zhanye Liu, Jianguang Xiao, Donghua Zhang, Xiaohua Yin

**Affiliations:** 1 Department of Respiratory and Critical Care Medicine, Linyi People's Hospital, Linyi, Shandong Province, China; 2 Department of Thoracic Surgery, Cao County People’s Hospital, Heze, Shandong Province, China; 3 Department of Thoracic Surgery, Laizhou People’s Hospital, Laizhou, Shandong Province, China; 4 Department of Oncology, Zhangqiu People’s Hospital, Zhangqiu, Shandong Province, China; 5 Department of Traditional Chinese Medicine, Shengli Oil Field Central Hospital, Dongying, Shandong Province, China

**Keywords:** NSCLC, progression, miR-320d, exosome, lncRNA LINC00662

## Abstract

Non-small cell lung cancer (NSCLC) is the most common tumor affecting modern people and is associated with severe morbidity and high mortality. Exosomal long non-coding RNAs as crucial regulators are involved in cancer progression. However, the role of exosomal lncRNA LINC00662 in the development of NSCLC remains unclear. Here, we aimed to explore the impact of exosomal lncRNA LINC00662 on the NSCLC progression and the underlying mechanism. Significantly, we revealed that the expression of lncRNA LINC00662 was elevated in the plasma exosome of NSCLC patients. Exosomal LINC00662 promoted proliferation, invasion, and migration, and inhibited apoptosis and cell cycle arrest of NSCLC cells. Mechanically, LINC00662 was able to serve as a miR-320d sponge in NSCLC cells. MiR-320d could target E2F1 in NSCLC cells. Exosomal LINC00662 contributed to the progression of NSCLC by miR-320d/E2F1 axis *in vitro*. Remarkably, exosomal LINC00662 enhanced the tumor growth of NSCLC *in vivo*. Thus, we conclude that exosomal lncRNA LINC00662 promotes NSCLC progression by modulating miR-320d/E2F1 axis. Our finding provides new insights into the mechanism by which exosomal lncRNA LINC00662 contributes to the development of NSCLC. LncRNA LINC00662, miR-320d, and E2F1 may serve as potential targets for NSCLC therapy.

## INTRODUCTION

Lung cancer serves as the most prevailing malignancy and is the principal cause of cancer-associated mortality globally, according to the latest annual statistics report of global cancer [1]. Non-small cell lung cancer (NSCLC) makes up about 83% of primary lung cancer [2]. Although the surgical and chemotherapeutic interventions have been advanced, the 5-year survival rate of NSCLC patients remains unsatisfactory, and the recurrence incidence of NSCLC patients is high due to drug resistance or tumor metastasis [3]. Understanding the mechanism underlying NSCLC development is critical for the diagnosis, therapy, and prognosis for NSCLC [4]. However, the advancement in this research field is still limited.

Long non-coding RNAs (lncRNAs) as the emerging essential biological modulator, play crucial roles in many physiological and pathological processes such as cell cycle, differentiation, apoptosis, cardiovascular diseases, and cancer progression [5, 6]. Many lncRNAs are involved in the modulation of NSCLC progression. For example, lncRNA-HIT increases NSCLC cell proliferation by affecting the expression of E2F1 [7]. LncRNA MALAT1 promotes the progression of NSCLC by regulating miR-200a-3p/programmed death-ligand-1 signaling [8]. The role of lncRNA LINC00662 in cancer development has been widely investigated [9]. It has been reported that lncRNA LINC00662 is involved in the modulation of lung cancer stem cells [10]. However, the effect of lncRNA LINC00662 on NSCLC progression is still unreported. Moreover, the nano-sized particles called exosome serves as transport vesicles of biological loads, such as miRNAs, mRNAs, proteins, and lncRNA, leading to the phenotypic impact on the receiver cells [11]. Circulating exosomes, loaded with regulative lncRNAs, display a critical function in long-distance cell communication, participating in cancer development [12]. It has been identified that the particular lncRNAs can be packaged into exosomes and are closely associated with the cancer pathogenesis and the clinical outcomes, which are highly correlated with the clinicopathological characteristics of cancer and thus may function as meaningful biomarkers [13]. Nevertheless, whether the lncRNA LINC00662 is packed in exosome and the role of exosomal lncRNA LINC00662 in the modulation of NSCLC remains elusive.

MicroRNAs (miRNAs) are described as short non-coding RNAs with a length of approximately 20-25 nucleotides and are involved in the modulation of numerous biological processes [14]. MiRNAs are able to control gene expression in the post-transcriptional levels by pairing with target mRNAs at the 3′ untranslated region (3′ UTR [14]. MiRNAs modulate different targets that hold essential functions in a broad spectrum of biological and medical processes, including cell apoptosis, proliferation, differentiation, invasion, metastasis, and tumorigenesis [15]. A substantial number of investigations have revealed that miRNAs are involved in the progression of NSCLC [16, 17]. Meanwhile, previous investigations identified that miR-320d played crucial roles in modulating tumorigenesis by interacting with the targeted genes [18]. In addition, the E2F transcription factor 1 (E2F1) serves as an essential transcription factor that is involved in diverse critical biological processes [19]. The abnormal elevation of E2F1 is observed in different types of human cancers and is associated with poor survival prognosis and malignant progress [20, 21]. E2F1 is involved in the modulation of NSCLC [22]. However, the correlation of miR-320d and E2F1 with lncRNA LINC00662 in the modulation of NSCLC is still unclear.

In this study, we aimed to explore the role and the underlying mechanism of exosomal lncRNA LINC00662 in the development of NSCLC. We identified a novel function of exosomal lncRNA LINC00662 in promoting NSCLC progression by regulating miR-320d/E2F1 axis.

## RESULTS

### The expression of lncRNA LINC00662 is elevated in the plasma exosome of NSCLC patients

**Figure 1:**
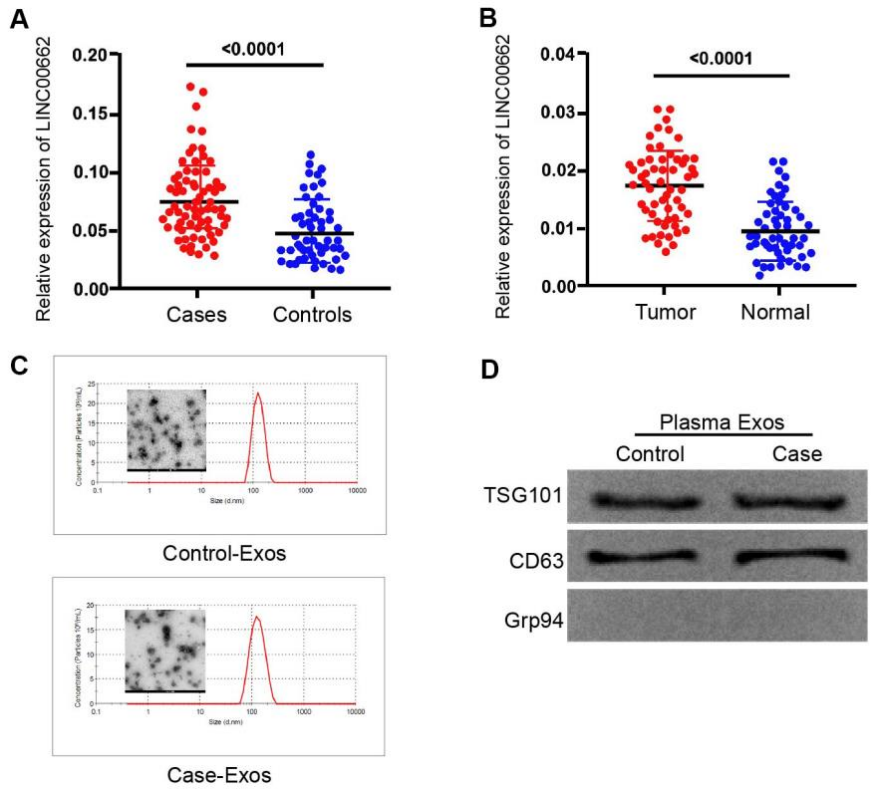
**The expression of lncRNA LINC00662 is elevated in the plasma exosome of NSCLC patients.** (**A**) The expression levels of lncRNA LINC00662 were measured by qPCR in the plasma exosome from NSCLC patients (n=50). (**B**) The expression levels of lncRNA LINC00662 were measured by qPCR in the NSCLC patient tissues (n=50) and the adjacent normal tissues (n=50). (**C**) The characteristics of exosomes were analyzed by the transmission electron microscopy (TEM) in the NSCLC patients. (**D**) The expression of TSG101, CD63, and Grp94 was tested by Western blot analysis in the exosome from NSCLC patients.

To assess the potential correlation of exosomal lncRNA LINC00662 with the NSCLC progression, we analyzed their expression in the plasma exosome of NSCLC patients. Significantly, the expression levels of LINC00662 were elevated in the plasma exosome from NSCLC patients (n=50) compared to those from normal cases (n=50) (Figure 1A). Besides, the expression levels of LINC00662 were up-regulated in the NSCLC patient tissues (n=50) compared to the adjacent normal tissues (n=50) (Figure 1B), implying that LINC00662 may be associated with the clinical development of NSCLC. Moreover, the transmission electron microscopy (TEM) showed that exosomes from the NSCLC patients presented the same sizes as those from the normal cases (Figure 1C). Western blot analysis revealed the existence of the exosome markers, including TSG101 and CD63, in the exosome of NSCLC patients and normal cases (Figure 1D).

### Exosomal LINC00662 promotes proliferation and inhibits apoptosis of NSCLC cells

**Figure 2:**
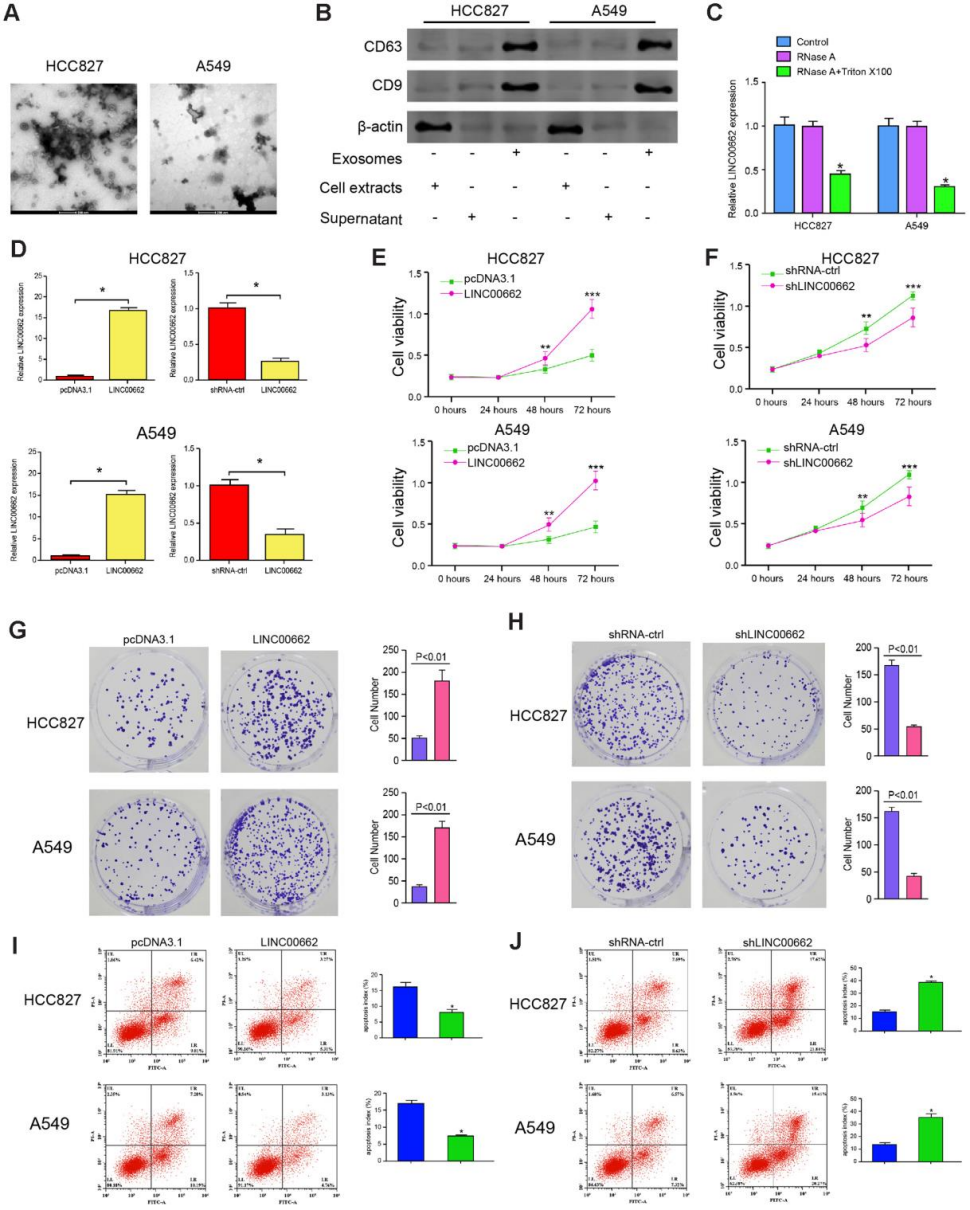
**Exosomal LINC00662 promotes proliferation and inhibits apoptosis of NSCLC cells.** (**A**) The characteristics of exosomes were analyzed by the transmission electron microscopy (TEM) in the HCC827 and A549 cells. (**B**) The expression of CD9 and CD63 was measured by the Western blot analysis in the exosome of the HCC827 and A549 cells. (**C**) The expression of LINC00662 was tested by qPCR in the HCC827 and A549 cells treated with RNase A (1 μg/mL) or co-treated with RNase A (1 μg/mL) and Triton X100 (0.1%). (**D**–**J**) The HCC827 and A549 cells were infected with the lentiviral plasmids carrying LINC00662 shRNA (shLINC00662) or corresponding control shRNA (shNC), or transfected with the LINC00662 overexpression vector or the corresponding control vector, and the exosomes were extracted and further treated the cells. (**D**) The efficiency of the LINC00662 depletion and the LINC00662 overexpression was validated by qPCR assays in the cells. (**E**, **F**) The cell viability was analyzed by the MTT assays in the cells. (**G**, **H**) The cell proliferation was measured by the colony formation assays in the cells. (**I**, **J**) The cell apoptosis was measure by flow cytometry analysis in the cells. Data are presented as mean ± SD. Statistic significant differences were indicated: * *P* < 0.05, ** *P* < 0.01.

Then, we further explored the effect of exosomal lncRNA LINC00662 on the progression of NSCLC *in vitro*. The exosomes were isolated from the culture medium of HCC827 and A549 cells and the characteristics were identified by TEM (Figure 2A). In addition, the expression of the exosome markers, including CD9 and CD63 was enriched in the exosomes (Figure 2B). Then, the expression of LINC00662 was measured in culture medium treated with RNase A or co-treated with RNase A and Triton X-100. Our data showed that the expression of LINC00662 was unacted on the treatment of RNase A while remarkably reduced upon the simultaneous co-treatment of RNase A and Triton X-100, indicating that extracellular LINC00662 was packaged in the membranes (Figure 2C). To evaluate the effect of LINC00662 on the progression of NSCLC *in vitro*, the HCC827 and A549 cells were infected with the lentiviral plasmids carrying LINC00662 shRNA (shLINC00662) or corresponding control shRNA (shNC), or transfected with the LINC00662 overexpression vector or the corresponding control vector, and the exosomes were extracted and further treated the cells. The efficiency of the LINC00662 depletion and the LINC00662 overexpression was validated in the cells (Figure 2D). MTT assays revealed that the overexpression of LINC00662 enhanced while the depletion of LINC00662 reduced the cell viability of the HCC827 and A549 cells (Figure 2E, 2F). Similarly, the LINC00662 overexpression increased the colony formation and the LINC00662 knockdown decreased this phenotype in the cells (Figure 2G, 2H). Moreover, cell apoptosis was inhibited by the overexpression of LINC00662 while promoted by the depletion of LINC00662 in the cells (Figure 2I, 2J). Together these data suggest that exosomal LINC00662 promotes proliferation and inhibits apoptosis of NSCLC cells.

### Exosomal LINC00662 promotes invasion and migration, and inhibits cell cycle arrest of NSCLC cells

**Figure 3:**
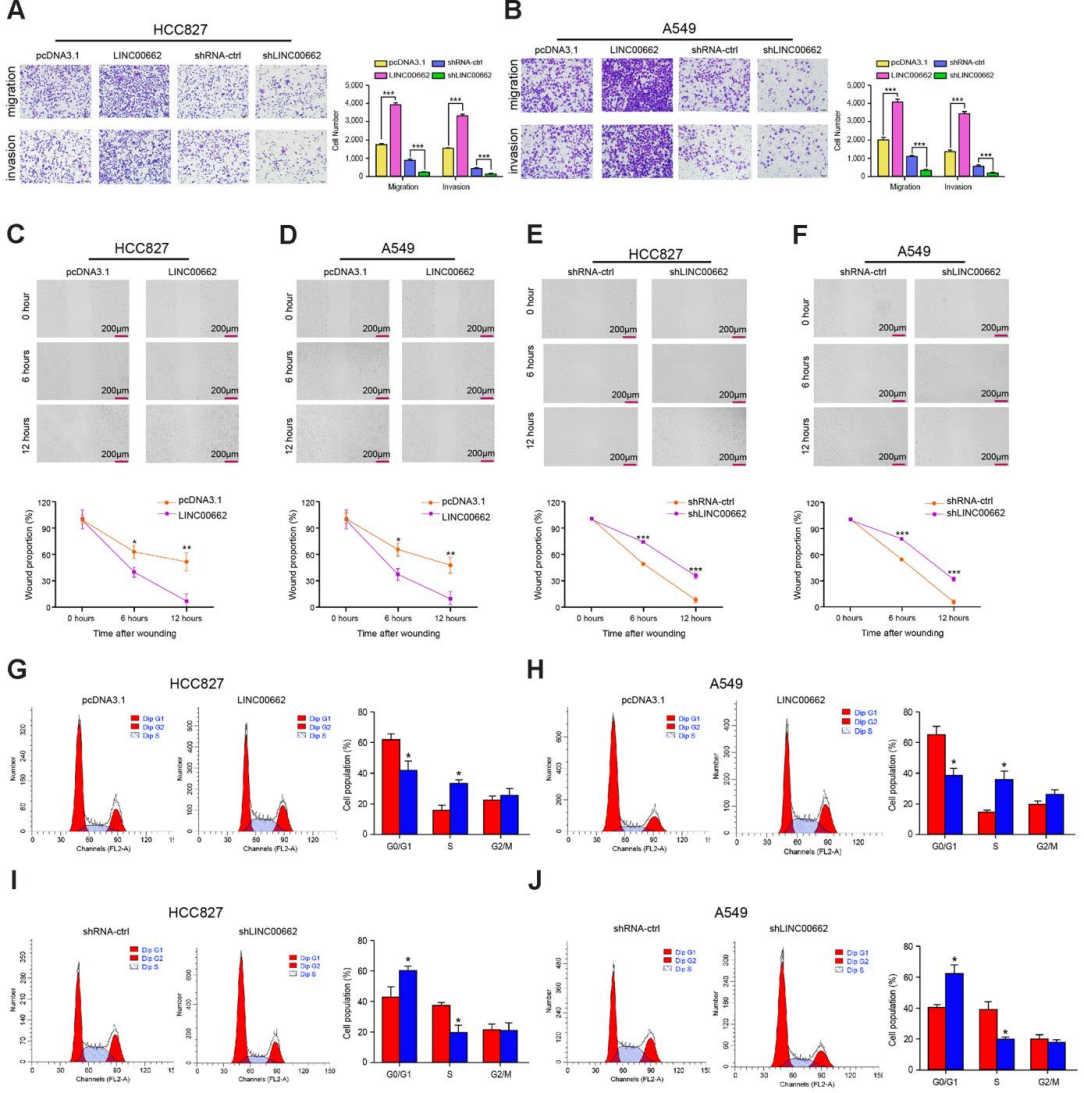
**Exosomal LINC00662 promotes invasion and migration of NSCLC cells.** (**A**–**F**) The HCC827 and A549 cells were infected with the lentiviral plasmids carrying LINC00662 shRNA (shLINC00662) or corresponding control shRNA (shNC), or transfected with the LINC00662 overexpression vector or the corresponding control vector, and the exosomes were extracted and further treated the cells. (**A**, **B**) The cell migration and invasion were examined by transwell assays in the cells. (**C**–**F**) The migration and invasion were measured by wound healing assays in the cells. The wound healing proportion was shown. (**G**–**J**) The cell cycle was analyzed by flow cytometry analysis in the cells. Data are presented as mean ± SD. Statistic significant differences were indicated: * *P* < 0.05, ** *P* < 0.01.

Then, we investigated the role of exosomal lncRNA LINC00662 in modulating the migration and invasion of NSCLC cells. Transwell assays revealed that the migration and invasion of HCC827 and A549 cells were significantly enhanced by the overexpression of exosomal lncRNA LINC00662 while were reduced by the depletion of exosomal lncRNA LINC00662 (Figure 3A, 3B). Similarly, the LINC00662 overexpression remarkably decreased the wound healing proportion, and the LINC00662 knockdown presented the reverse results in the cells (Figure 3C–3F), suggesting that exosomal lncRNA LINC00662 contributes to the migration and invasion of NSCLC cells *in vitro*. Moreover, the G0/G1 phase cells were reduced while the S phase cells were enhanced by the overexpression of LINC00662 in the cells, but the depletion of exosomal lncRNA LINC00662 showed a reversed effect (Figure 3G–3J), suggesting that exosomal lncRNA LINC00662 was able to inhibit cell cycle arrest at the G0/G1 phase in the NSCLC cells.

### LINC00662 serves as a miR-320d sponge in NSCLC cells

**Figure 4:**
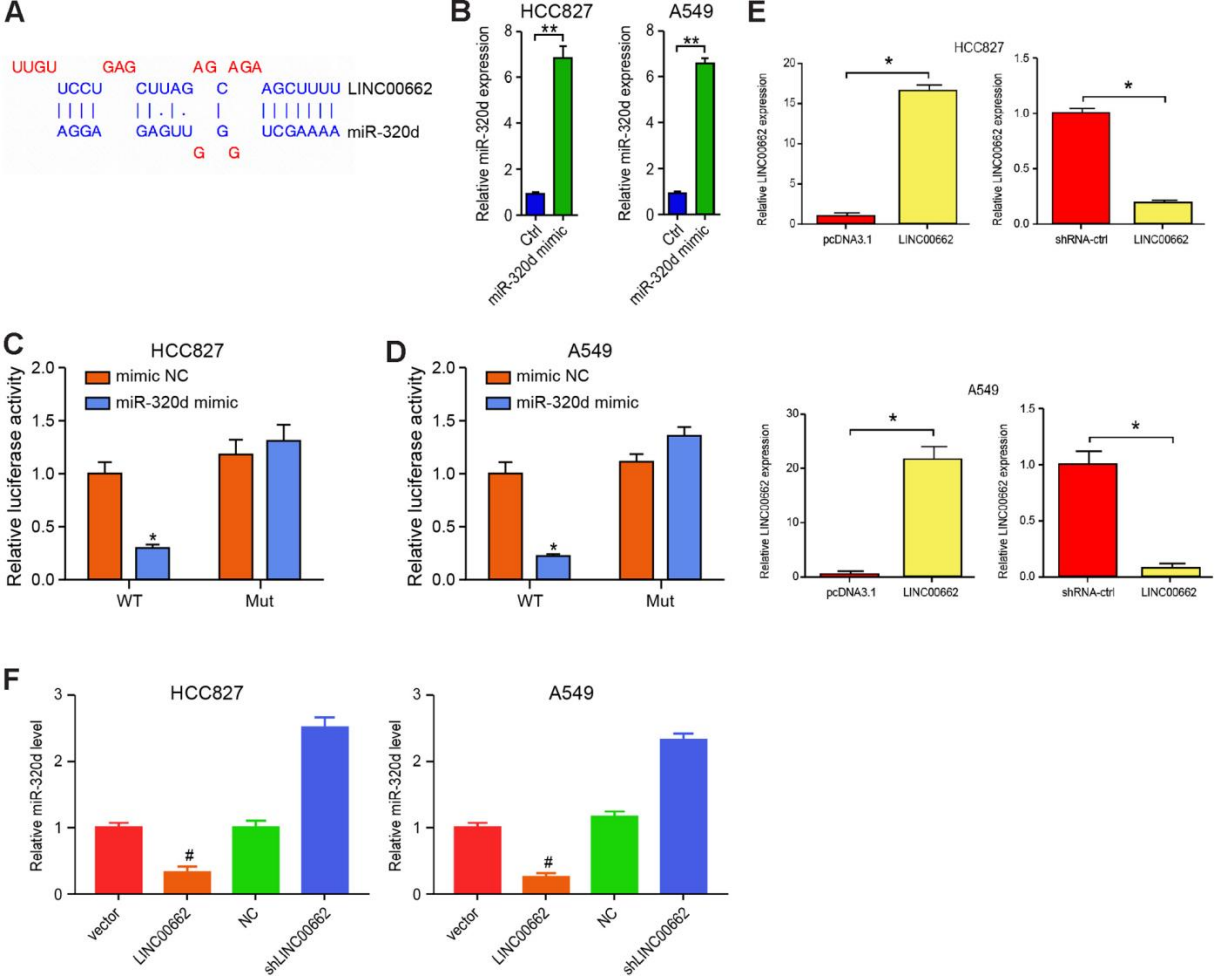
**LINC00662 serves as a miR-320d sponge in NSCLC cells.** (**A**) Potential interaction between lncRNA LINC00662 and miR-320d was identified by the bioinformatic analysis using ENCORI (http://starbase.sysu.edu.cn/index.php). (**B**) The expression levels of miR-320d were tested by qPCR in the HCC827 and A549 cells treated with control mimic (miR-NC) or miR-320d mimics. (**C**, **D**) Luciferase activities of LINC00662 (LINC00662 WT) and LINC00662 with the miR-320d-binding site mutant (LINC00662 MUT) were determined by luciferase reporter gene assays in the HCC827 and A549 cells treated with control mimic (miR-NC) or miR-320d mimic. (**E**) The efficiency of the LINC00662 depletion and the LINC00662 overexpression was validated by qPCR assays in the cells. (**F**) The HCC827 and A549 cells were treated with the lentiviral plasmids carrying LINC00662 shRNA (shLINC00662) or corresponding control shRNA (shNC), or transfected with the LINC00662 overexpression vector or the corresponding control vector. The expression of miR-320d was tested by qPCR assays in the cells. Data are presented as mean ± SD. Statistic significant differences were indicated: * *P* < 0.05, ** *P* < 0.01.

Next, we tried to explore the mechanism of exosomal lncRNA LINC00662-mediated NSCLC progression. We identified the potential interaction between lncRNA LINC00662 and miR-320d in the bioinformatics analysis by using ENCORI (http://starbase.sysu.edu.cn/index.php) (Figure 4A). Then, we treated the HCC827 and A549 cells with miR-320d mimic or the corresponding control mimic, and the efficiency was verified in the cells (Figure 4B). The miR-320d mimic remarkably reduced the luciferase activities of LINC00662 but failed to affect the LINC00662 with the miR-320d-binding site mutant in the cells (Figure 4C, 4D). The efficiency of the LINC00662 depletion and the LINC00662 overexpression was validated in the cells (Figure 4E). The LINC00662 overexpression reduced while the LINC00662 depletion enhanced the expression of miR-320d in the cells (Figure 4F). Together these suggest that LINC00662 serves as a miR-320d sponge in NSCLC cells.

### MiR-320d targets E2F1 in NSCLC cells

**Figure 5:**
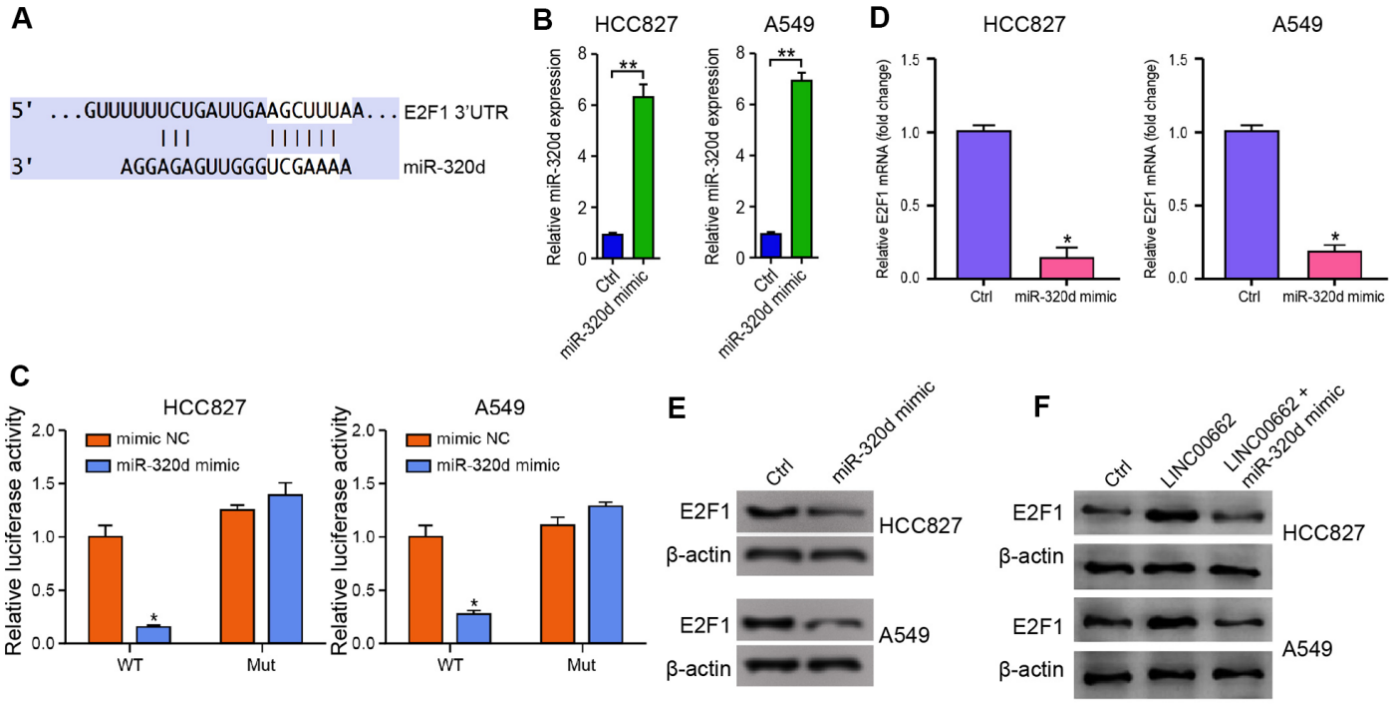
**MiR-320d targets E2F1 in NSCLC cells.** (**A**) The interaction of miR-320d and E2F1 3’ UTR was identified by bioinformatic analysis using Targetscan (http://www.targetscan.org/vert_72/). (**B**–**E**) The HCC827 and A549 cells were treated with miR-320d mimic or the control mimic. (**B**) The expression of miR-320d was tested by qPCR assays in the cells. (**C**) The luciferase activities of wild type E2F1 (E2F1 WT) and E2F1 with the miR-320d-binding site mutant (E2F1 MUT) were determined by luciferase reporter gene assays in the cells. (**D**) The mRNA expression of E2F1 was measured by qPCR assays in the cells. (**E**) The protein expression of E2F1 was analyzed by Western blot analysis in the cells. (**F**) The HCC827 and A549 cells were treated with the control vector, LINC00662 overexpression vector, or co-treated with LINC00662 overexpression vector and miR-320d mimic. The protein expression of E2F1 was measured by Western blot analysis in the cells. Data are presented as mean ± SD. Statistic significant differences were indicated: * *P* < 0.05, ** *P* < 0.01.

Then, we identified the miR-320d-targeted site in E2F1 3’ UTR in a bioinformatic analysis by using Targetscan (http://www.targetscan.org/vert_72/) (Figure 5A). To determine the impact of miR-320d on E2F1, the HCC827 and A549 cells were treated with miR-320d mimic, and the efficiency was validated in the cells (Figure 5B). Notably, the miR-320d mimic treatment inhibited the luciferase activities of wild type E2F1but failed to affect the E2F1 with the miR-320d-binding site mutant in the cells (Figure 5C). Furthermore, the mRNA and protein expression of E2F1 were significantly decreased by miR-320d mimic in the cells (Figure 5D, 5E), suggesting that miR-320d is able to target E2F1 in the NSCLC cells. Moreover, the expression of E2F1 was enhanced by the overexpression of LINC00662, in which the miR-320d mimic could block this enhancement in the cells (Figure 5F).

### Exosomal LINC00662 promotes the progression of NSCLC by miR-320d/E2F1 axis *in vitro*

**Figure 6:**
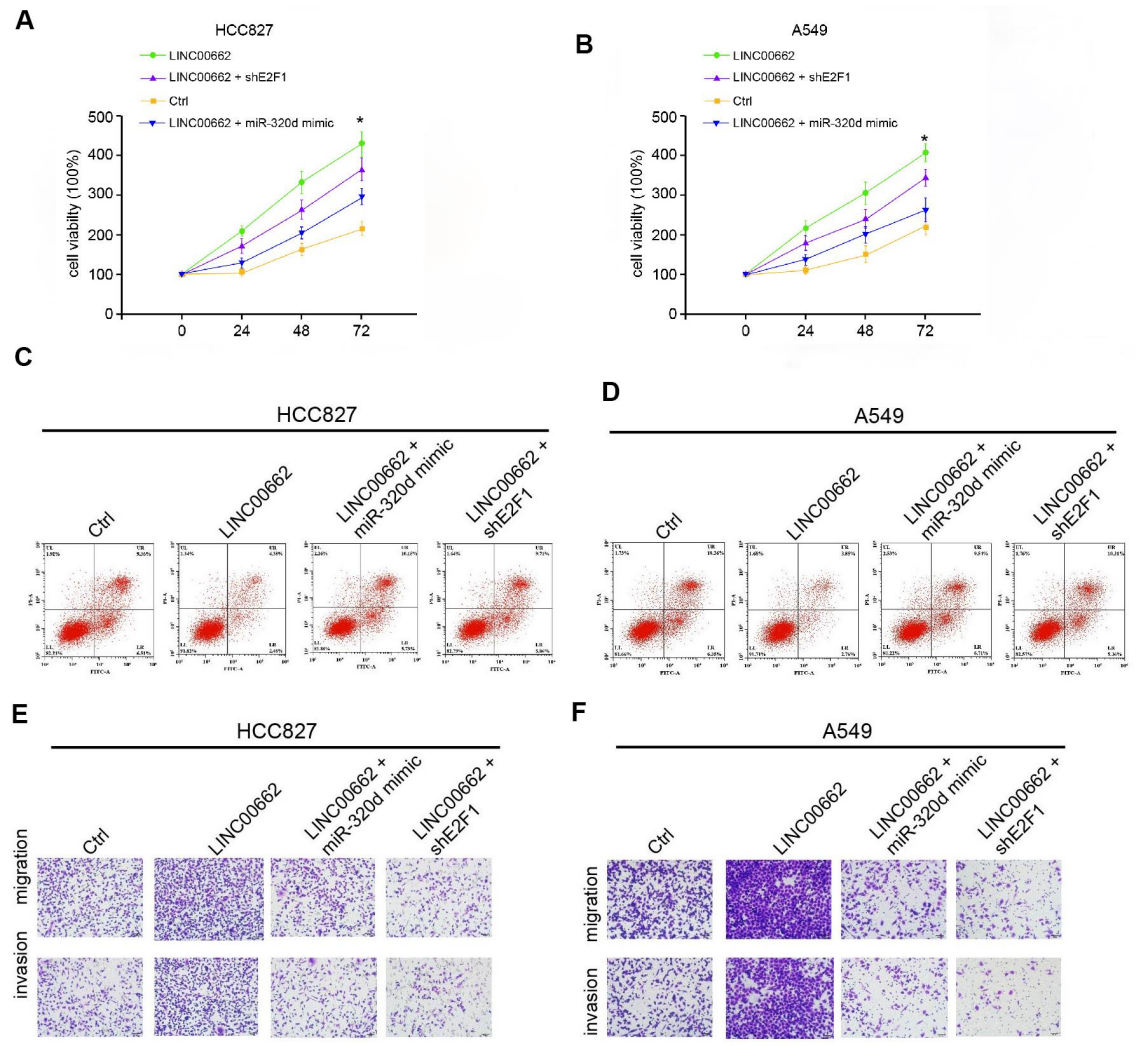
**Exosomal LINC00662 promotes the progression of NSCLC by miR-320d/E2F1 axis *in vitro*.** (**A**–**F**) The HCC827 and A549 cells were treated with LINC00662 overexpression vector, or co-treated with LINC00662 overexpression vector and miR-320d mimic or lentiviral plasmids carrying E2F1 shRNA. (**A**, **B**) The cell viability was analyzed by the MTT assays in the cells. (**C**, **D**) The cell apoptosis was measure by flow cytometry analysis in the cells. (**E**, **F**) The cell migration and invasion were examined by transwell assays in the cells. Data are presented as mean ± SD. Statistic significant differences were indicated: * *P* < 0.05, ** *P* < 0.01.

We then explored the role of the exosomal LINC00662/miR-320d/E2F1 axis in NSCLC development *in vitro*. MTT assays revealed that the exosomal overexpression of LINC00662 enhanced the cell viability of HCC827 and A549 cells, in which the miR-320d mimic or the E2F1 depletion by shRNA could block this enhancement (Figure 6A, 6B). The miR-320d mimic or the E2F1 knockdown rescued the cell apoptosis inhibited by the exosomal LINC00662 overexpression in the cells (Figure 6C, 6D). Transwell assays revealed that the migration and invasion of HCC827 and A549 cells were significantly promoted by the overexpression of exosomal lncRNA LINC00662, in which the miR-320d mimic or the E2F1 depletion could attenuate this phenotype (Figure 6E, 6F). Together these indicate that exosomal LINC00662 promotes the progression of NSCLC by miR-320d/E2F1 axis *in vitro*.

### Exosomal LINC00662 contributes to the tumor growth of NSCLC *in vivo*

**Figure 7:**
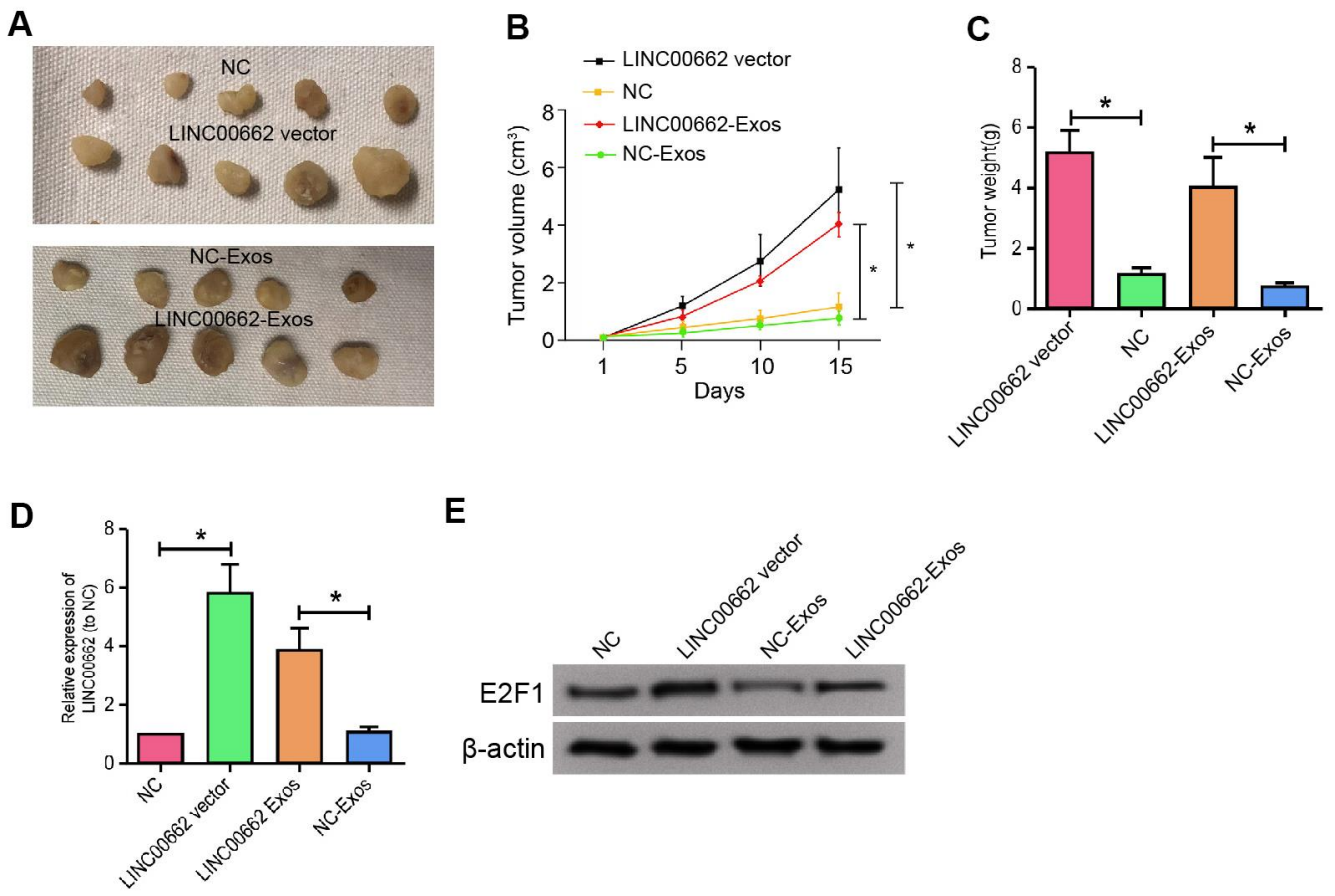
**Exosomal LINC00662 contributes to the tumor growth of NSCLC *in vivo*.** (**A**–**D**) The effect of exosomal LINC00662 on tumor growth of NSCLC cells *in vivo* was analyzed by nude mice tumorigenicity assay. The A549 cells were treated with LINC00662 overexpression vector or LINC00662 overexpression exosome. (**A**) Representative images of dissected tumors from nude mice were presented. (**B**) The average tumor volume was calculated and shown. (**C**) The average tumor weight was calculated and shown. (**D**, **E**) The protein expression levels of E2F1 and β-actin were examined by Western blot analysis in the tumor tissues. Data are presented as mean ± SD. Statistic significant differences were indicated: * *P* < 0.05, ** *P* < 0.01.

We further determined the impact of exosomal LINC00662 on the NSCLC development *in vivo*. For this purpose, we performed the tumorigenicity analysis in nude mice injected with A549 cells, which were treated with LINC00662 overexpression vector or LINC00662 overexpression exosome. The overexpression of LINC00662 significantly enhanced the tumor growth of A549 cells *in vivo*, as demonstrated by the tumor size (Figure 7A), tumor volume (Figure 7B), and tumor weight (Figure 7C). Besides, the expression of E2F1 was increased by the LINC00662 overexpression in the tumor tissues of the mice (Figure 7D, 7E). Together these suggest that exosomal LINC00662 contributes to the tumor growth of NSCLC *in vivo*.

## DISCUSSION

NSCLC is the predominant type of lung cancer with severe morbidity and high mortality [23]. As a crucial regulator in cancer development, exosomal lncRNAs are widely identified in the modulation of NSCLC. It has been reported that the exosome-regulated shift of lncRNA RP11 838N2.4 elevates the resistance of erlotinib in NSCLC [24]. Cancer-originated exosome lncRNA GAS5 serves as a marker of diagnosis of NSCLC [25]. Plasma lncRNA MALAT-1 protected by exosome is elevated and increases cell migration and proliferation in NSCLC [26]. Tumor-transmitted lncRNA H19 stimulates gefitinib resistance by packaging in the exosome of NSCLC [27]. Exosome-transmitted lncRNA UFC1 enhances the progression of NSCLC through EZH2-regulated epigenetic inhibiting of PTEN expression [28]. Moreover, it has been identified that lncRNA LINC00662 contributes to the development of multiple cancers, such as breast cancer, ovarian cancer, and colorectal cancer [29–31]. In this study, we first identified that lncRNA LINC00662 was elevated in the plasma exosome of NSCLC patients. Exosomal LINC00662 promoted proliferation, invasion, and migration, and inhibited apoptosis of NSCLC cells. These data present a novel function of exosomal lncRNA LINC00662 in the NSCLC progression, providing valuable evidence for the fundamental role of exosomal lncRNAs in the development of NSCLC.

As a primary component of non-coding RNA and the significant interplay factors with lncRNAs in the pathological processes, miRNAs are also involved in the modulation of NSCLC. It has been reported that microRNA-455 represses NSCLC by targeting ZEB1 [32]. MiR-451a decreases cell invasion and migration in NSCLC by modulating the expression of ATF2 [33]. MiR-24 increases the invasion and migration of NSCLC by regulating ZNF367 [34]. MiRNA-4735-3p-mediated FBXL3 inhibits cell proliferation and migration of NSCLC [35]. MiR-760 represses the proliferation and metastasis of NSCLC by controlling the expression of ROS1 [36]. MiR-24 increases the invasion and migration of NSCLC by regulating ZNF367 [34]. Besides, the role of miR-320d in the modulation of development of cancer has been identified in several tumor models, including hepatocellular carcinoma, gastric cardiac adenocarcinoma, and colorectal cancer [37–39]. Our mechanical investigation further demonstrated that LINC00662 served as a miR-320d sponge and miR-320d targeted E2F1 in the NSCLC cells. These data display an unreported role of miR-320d in the development of NSCLC, identifying the new upstream lncRNA LINC00662 and downstream target E2F1 of miR-320d in the modulation of NSCLC.

It has been identified that E2F1 is involved in the development of NSCLC. E2F1 positively controls the expression of interferon regulatory factor 5 in NSCLC [40]. PAQR4 stimulates cell metastasis and proliferation by the CDK4/pRB/E2F1 signaling in NSCLC [41]. MiR-1205 serves as a cancer suppressor by disengaging the interplay of MDM4/E2F1 and KRAS in NSCLC [42]. LncRNA-HAGLR represses tumor growth of NSCLC by epigenetically inhibiting E2F1 [43]. The clinical significance of the protein expression of E2F1 in NSCLC is identified [44]. Our data showed that E2F1 contributed to the progression of NSCLC and is targeted by miR-320d, which could be sponged by lncRNA LINC00662 in the system. These data provide new evidence that E2F1 serves as a crucial factor in the development of NSCLC.

In conclusion, we discovered that exosomal lncRNA LINC00662 promoted NSCLC progression by modulating miR-320d/E2F1 axis. Our finding provides new insights into the mechanism by which exosomal lncRNA LINC00662 contributes to the development of NSCLC, improving the understanding of exosomal lncRNA LINC00662 and NSCLC. LncRNA LINC00662, miR-320d, and E2F1 may serve as potential targets for NSCLC therapy.

## MATERIALS AND METHODS

### NSCLC clinical samples

A total of 50 NSCLC clinical samples used in this study was obtained between June 2017 and February 2019 from Linyi People's Hospital. The characteristic information of the NSCLC patients was listed in Supplementary Table 1. All the patients were diagnosed by histopathological analysis. All cases were independently diagnosed and reviewed by two clinicians. Before surgery, no systemic or local therapy was carried out in the subjects. The NSCLC tissues and corresponding para-neoplastic tissues obtained from the patients were immediately frozen into the liquid nitrogen, followed by storing at -80° C before further analysis. The samples used in this study were under the written approval of the patients and healthy cases. This study conformed to the experimental guidelines of the World Medical Association and the Ethics Committee of Linyi People's Hospital.

### Exosome isolation and analysis

The culture medium and plasma and were centrifuged (3000 × g, 15 minutes) to drop cells and cellular debris. Next, Exoquick exosome precipitation solution (System Biosciences, USA) was utilized to isolate exosomes. The exosome characteristics were analyzed by the transmission electron microscopy (TEM) as previously reported [45].

### Cell culture and treatment

The HCC827 and A549 cell lines were purchased in American Type Tissue Culture Collection. The cells were cultured in the medium of DMEM (Gibco, USA) containing 10% fetal bovine serum (Gibco, USA), 0.1 mg/mL streptomycin (Gibco, USA) and 100 units/mL penicillin (Gibco, USA) at a condition of 37° C with 5% CO_2_. The LINC00662 overexpression vector and control vector, LINC00662 shRNA and control shRNA, E2F1 shRNA and control siRNA, miR-320d mimic and control mimic were obtained (GenePharma, China). The transfection in the cells was performed by Liposome 3000 (Invitrogen, USA) according to the manufacturer's instructions.

### Quantitative reverse transcription-PCR (qRT-PCR)

The total RNAs were extracted by TRIZOL (Invitrogen, USA) from the tissues and cells. The first-strand cDNA was synthesized using Stand cDNA Synthesis Kit (Thermo, USA) as the manufacturer's instruction. The qRT-PCR was carried out by applying SYBR Real-time PCR I kit (Takara, Japan). The standard control for mRNA/lncRNA and miRNA was GAPDH and U6, respectively. Quantitative determination of the RNA levels was conducted by SYBR GreenPremix Ex TaqTM II Kit (TaKaRa, Japan). The experiments were independently repeated at least three times. The primer sequences are as follows:

LncRNA LINC00662 forward: 5′-CACGCTTCTGAAACTGGTGT-3′

LncRNA LINC00662 reverse: 5′-TGTACAGCCTGGTGACAGAG-3′

miR-320d forward: 5′-AAAAGCTGGGTTGAGAGGA-3′

miR-320d reverse: 5′-TCCTCTCAACCCAGCTTTT-3′

E2F1 forward: 5′-AGCGGCGCATCTATGACATC-3′

E2F1 reverse: 5′-GTCAACCCCTCAAGCCGTC-3′

GAPDH forward: 5′-AAGAAGGTGGTGAAGCAGGC-3′.

GAPDH reverse: 5′-TCCACCACCCAGTTGCTGTA-3′

U6 forward: 5′-GCTTCGGCAGCACATATACTAA-3′

U6 reverse: 5′-AACGCTTCACGAATTTGCGT-3′

### MTT assays

The cell viability was measured by MTT assays. Briefly, about 2×10^4^ cells were put into 96 wells and cultured for 12 hours. After indicated treatment for 24 hours, 48 hours, and 72 hours, the cells were added with a 10 μL MTT solution (5 mg/mL) and cultured for an extra 4 hours. Discarded medium, and 150 μL DMSO was used to treat the wells. An ELISA browser was applied to analyze the absorbance at 570nm (Bio-Tek EL 800, USA).

### Colony formation assays

About 1×10^3^ HCC827 and A549 cells were layered in 6 wells and incubated in DMEM at 37° C. After two weeks, cells were cleaned with PBS Buffer, made in methanol for about thirty minutes, and dyed with crystal violet dye at the dose of 1%, after which the number of colonies was calculated.

### Transwell assays

Transwell assays analyzed the impacts of the exosomal lncRNA LINC00662 on cell invasion and migration of NSCLC by using a Transwell plate (Corning, USA) according to the manufacturer's instruction. Briefly, the upper chambers were plated with around 1 × 10^5^ cells. Then solidified through 4% paraformaldehyde and dyed with crystal violet. The invaded and migrated cells were recorded and calculated.

### Wound healing assay

HCC827 and A549 cells were plated in the 24-well plate at 3 × 10^5^/well and cultured overnight to reach a full confluent as a monolayer. A 20μl pipette tip was applied to slowly cut a straight line across the well. Then the well was washed by PBS 3 times and changed with the serum-free medium and continued to culture. The wound healing percentage was calculated.

### Analysis of cell apoptosis

Around 2×10^5^ cells were plated on 6-well dishes. Cell apoptosis was analyzed by using the Annexin V-FITC Apoptosis Detection Kit (CST, USA) according to the manufacture’s instruction. Briefly, about 2×10^6^ collected and washed cells collected by binding buffer and were dyed at 25° C, followed by the flow cytometry analysis.

### Cell-cycle analysis

Approximately 1×10^5^ cells were plated on 6-well dishes and treated as indicated. Floating and adherent cells were fixed in cold ethanol (4° C, 70% in PBS) overnight. RNaseA 1(00 μg/mL) was added to the cells at 37° C for 30 minutes, followed by the PI staining (50 μg/mL, 30 minutes) in the dark and the flow cytometric analysis using a FACSCalibur cytometry (Becton Dickinson, USA). About ten thousand events were calculated for each sample and the distribution of cell cycle was analyzed by Cell Quest software (Becton Dickinson, USA).

### Luciferase reporter gene assay

The luciferase reporter gene assays were performed by using the Dual-luciferase Reporter Assay System (Promega, USA). Briefly, the cells were treated with the miR-320d mimic, miR-320d mimic or control mimic, pmirGLO-LINC00662, pmirGLO-LINC00662 mutant, pmirGLO-E2F1, and pmirGLO-E2F1 mutant were transfected in the cells by using Lipofectamine 3000 (Invitrogen, USA), followed by the analysis of luciferase activities, in which Renilla was applied as a normalized control.

### Western blot analysis

Total proteins were extracted from the cells or mice tissues with RIPA buffer (CST, USA). Protein concentrations were measured by using the BCA Protein Quantification Kit (Abbkine, USA). Same concentration of protein was divided by SDS-PAGE (12% polyacrylamide gels), transferred to PVDF membranes (Millipore, USA) in the subsequent step. The membranes were hindered with 5% milk and hatched overnight at 4° C with the primary antibodies for TSG101 (Abcam, USA), CD63 (Abcam, USA), Grp94 (Abcam, USA), CD9 (Abcam, USA), E2F1 (Abcam, USA), and β-actin (Abcam, USA), in which β-actin served as the control. Then, the corresponding second antibodies (Abcam, USA) were used for hatching the membranes 1 hour at room temperature, followed by the visualization by using an Odyssey CLx Infrared Imaging System.

### Analysis of tumorigenicity in nude mice

The effect of exosomal lncRNA LINC00662 on tumor growth *in vivo* was analyzed in nude mice of Balb/c (male, 4-week-old) (n=5). The A549 cells were treated with LINC00662 overexpression vector or LINC00662 overexpression exosome. And about 1×10^7^ cells were subcutaneously injected in the mice. After 7 days of injection, we measured tumor growth every 7 days. We sacrificed the mice after 28 days of injection, and tumors were scaled. Tumor volume (V) was observed by estimating the length and width with calipers and measured with the method × 0.5. The protein expression levels of E2F1 (Abcam, USA), and β-actin (Abcam, USA) were analyzed by Western blot analysis in the tumor tissues. Animal care and method procedure were authorized by the Animal Ethics Committee of Linyi People's Hospital.

### Statistical analysis

Data were presented as mean ± SD, and the statistical analysis was performed by GraphPad prism 7. The unpaired Student’s *t*-test was applied for comparing two groups, and the one-way ANOVA was applied for comparing among multiple groups. *P* < 0.05 were considered as statistically significant.

## Supplementary Material

Supplementary Table 1
