# Supplementary material for: Exosomal long non-coding RNA LINC00662 promotes non-small cell lung cancer progression by miR-320d/E2F1 axis
Source: Aging (Albany NY). 2021 Feb 11;13(4):6010–24. doi: 10.18632/aging.202522 (PMC7950287; doi:10.18632/aging.202522)
Supplement: Supplementary Table 1 [file aging-13-202522-s001.pdf]

SUPPLEMENTARY TABLE

Supplementary Table 1. The characteristics of NSCLC patients.

No.	Age	Gender	Organ	Smoking history	Tumor size (cm)	TNM stage
1	59	F	Lung	Non-smokers	<3	I–II
2	60	M	Lung	Smokers	≥3	III–IV
3	65	M	Lung	Smokers	<3	I–II
4	43	M	Lung	Smokers	≥3	III–IV
5	60	F	Lung	Non-smokers	≥3	III–IV
6	41	M	Lung	Smokers	<3	I–II+
7	45	M	Lung	Smokers	≥3	III–IV
8	56	M	Lung	Smokers	≥3	I–II-
9	70	M	Lung	Non-smokers	≥3	III–IV
10	67	M	Lung	Smokers	≥3	III–IV
11	59	M	Lung	Smokers	≥3	III–IV
12	57	M	Lung	Smokers	<3	I–II
13	61	F	Lung	Non-smokers	≥3	III–IV
14	51	F	Lung	Non-smokers	≥3	I–II
15	56	M	Lung	Smokers	≥3	III–IV
16	60	M	Lung	Smokers	≥3	III–IV
17	54	M	Lung	Smokers	≥3	III–IV
18	36	M	Lung	Smokers	≥3	I–II
19	46	M	Lung	Smokers	≥3	III–IV
20	60	M	Lung	Smokers	≥3	III–IV
21	59	M	Lung	Smokers	≥3	III–IV
22	57	F	Lung	Non-smokers	<3	I–II
23	51	M	Lung	Smokers	≥3	III–IV
24	38	M	Lung	Smokers	≥3	III–IV
25	56	M	Lung	Smokers	≥3	III–IV
26	49	M	Lung	Non-smokers	≥3	III–IV
27	58	F	Lung	Non-smokers	<3	I–II
28	46	M	Lung	Smokers	≥3	III–IV
29	56	M	Lung	Smokers	≥3	III–IV
30	54	M	Lung	Smokers	≥3	I–II
31	39	M	Lung	Smokers	<3	III–IV
32	53	M	Lung	Non-smokers	≥3	III–IV
33	60	M	Lung	Smokers	≥3	III–IV
34	66	M	Lung	Smokers	<3	I–II
35	60	F	Lung	Non-smokers	<3	I–II
36	59	M	Lung	Smokers	≥3	III–IV
37	69	M	Lung	Smokers	≥3	III–IV
38	38	M	Lung	Smokers	<3	I–II
39	56	M	Lung	Smokers	≥3	III–IV
40	53	F	Lung	Non-smokers	≥3	I–II
41	47	M	Lung	Smokers	≥3	III–IV
42	67	M	Lung	Non-smokers	<3	I–II
43	49	M	Lung	Smokers	≥3	III–IV
44	46	M	Lung	Non-smokers	≥3	III–IV
45	57	M	Lung	Smokers	<3	I–II
46	49	M	Lung	Smokers	<3r	I–II
47	47	M	Lung	Smokers	≥3	III–IV
48	62	M	Lung	Smokers	≥3	III–IV
49	68	F	Lung	Non-smokers	<3	I–II
50	66	M	Lung	Non-smokers	<3	III–IV
